# An appraisal of drug development timelines in the Era of precision oncology

**DOI:** 10.18632/oncotarget.10588

**Published:** 2016-07-13

**Authors:** Denis Leonardo Jardim, Maria Schwaederle, David S. Hong, Razelle Kurzrock

**Affiliations:** ^1^ Department of Clinical Oncology, Hospital Sirio Libanes, Sao Paulo, Brazil; ^2^ Center for Personalized Cancer Therapy and Division of Hematology and Oncology, University of California, San Diego, CA, USA; ^3^ Department of Investigational Cancer Therapeutics (Phase I Clinical Trials Program), The University of Texas MD Anderson Cancer Center, Houston, TX, USA

**Keywords:** drug development, precision medicine, pharmacoeconomics, biomarkers, FDA

## Abstract

The effects of incorporating a biomarker-based (personalized or precision) selection strategy on drug development timelines for new oncology drugs merit investigation. Here we accessed documents from the Food and Drug Administration (FDA) database for anticancer agents approved between 09/1998 and 07/2014 to compare drugs developed with and without a personalized strategy. Sixty-three drugs were included (28 [44%] personalized and 35 [56%] non-personalized). No differences in access to FDA-expedited programs were observed between personalized and non-personalized drugs. A personalized approach for drug development was associated with faster clinical development (Investigational New Drug [IND] to New Drug Application [NDA] submission; median = 58.8 months [95% CI 53.8–81.8] vs. 93.5 months [95% CI 73.9–112.9], *P* =.001), but a similar approval time (NDA submission to approval; median=6.0 months [95% CI 5.5–8.4] vs. 6.1 months [95% CI 5.9–8.3], *P* = .756) compared to a non-personalized strategy. In the multivariate model, class of drug stratified by personalized status (targeted personalized vs. targeted non-personalized vs. cytotoxic) was the only independent factor associated with faster total time of clinical drug development (clinical plus approval phase, median = 64.6 vs 87.1 vs. 112.7 months [cytotoxic], *P* = .038). Response rates (RR) in early trials were positively correlated with RR in registration trials (*r* = 0.63, *P* = <.001), and inversely associated with total time of drug development (*r* = −0.29, *P* = .049). In conclusion, targeted agents were developed faster than cytotoxic agents. Shorter times to approval were associated, in multivariate analysis, with a biomarker-based clinical development strategy.

## INTRODUCTION

Recent improvements in the understanding of cancer biology have posed new challenges for drug development in oncology. Traditional tumor histologies are being sliced into small molecular subsets, which are differentiated from each other based on specific targets driving tumor growth [1–3]. In addition, newly described genomic alterations are explaining the pathophysiology of rare tumors [4]. Consequently, new agents aiming to target these specific alterations are emerging and entering the drug development pathway.

The March 2006 Food and Drug Administration (FDA) critical path report stressed that the development of biomarkers and streamlining of clinical trials were major priorities for medical product development [5]. Incorporating biomarkers for treatment selection in cancer therapy holds the promise of enhancing efficacy of new treatments and improving the success of drug approvals [6]. Indeed, we previously showed that the incorporation of a biomarker-driven rationale for drug development (personalized/precision therapy strategy) was associated with improvements in response rate, progression-free and overall survival for FDA-approved anticancer agents [7].

Considering the fragmentation of classic tumor types into small subgroups and the recognition of new molecular targets of rare tumors to be included in new trials, it is expected that only small numbers of patients may be suitable for clinical trials using a biomarker-based approach. Hence, concerns about screen failures for biomarker-based trials and recruitment hurdles for more rare subsets of cancer exist, and theoretically may impact the length of the development process for these drugs [8, 9]. Nonetheless, the advancement of crizotinib to drug approval in only three years after reporting ALK rearrangement as a target in lung cancer [10], and the approval of ceritinib (a next generation ALK inhibitor) after phase I testing [11] suggests that a personalized approach is feasible and perhaps more efficient.

The FDA has developed special programs to improve drug approval timelines (Supplementary Table S1). One of the aims of these programs is to incentivize the development of agents designed to treat rare and life-threatening diseases, including molecular subtypes of advanced cancers. Both crizotinib and ceritinib were granted access to FDA-expedited programs, including accelerated approval. Nonetheless, a prior study failed to demonstrate faster drug development with expedited programs [12], questioning the promise of these policies. To date, little is known about the effects of incorporating a biomarker-based rationale for the development of new oncology drugs upon the length of drug development and the role of FDA programs in this new drug development paradigm. Herein, we performed a comprehensive analysis of the development process of FDA-approved drugs for advanced cancer between 09/1998 and 07/2014. We aimed to compare the timeline of drugs developed with or without a biomarker-based strategy and the influence of access to FDA expedited programs.

## RESULTS

### Search results and characteristics of drugs

**Figure 1:**
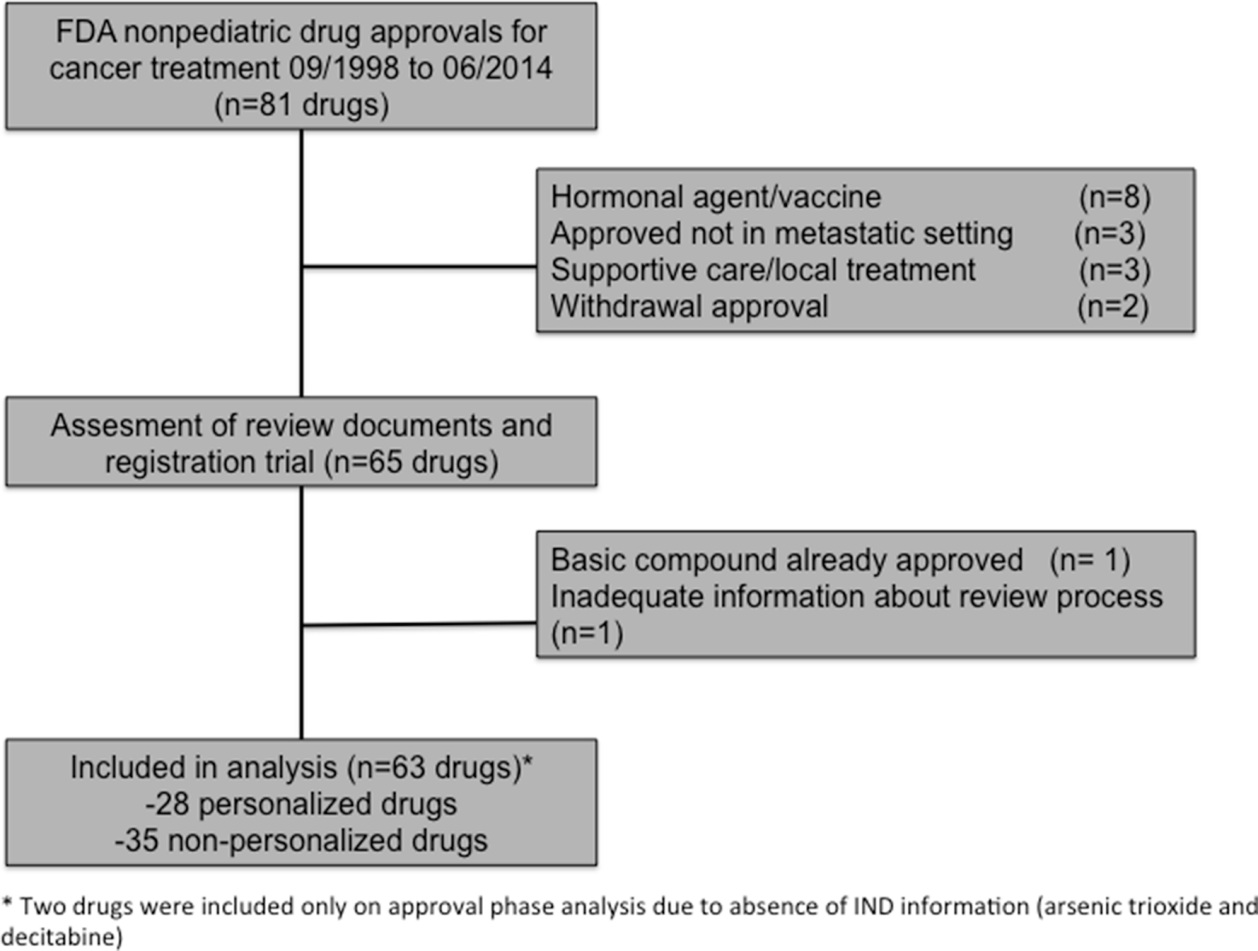
Flow chart of drug selection

We initially identified 81 FDA-approved drugs for adult cancer treatment indications and 63 drugs were selected for further analysis after applying selection criteria as described in the methods section (Figure 1). Twenty-eight drugs (44%) were developed in a personalized fashion and 35 (56%) in a non-personalized manner according to first NDA obtained (Supplementary Table S3). All personalized drugs were classified as targeted agents, while 21 (60%) of the non-personalized agents were targeted and 14 (40%) were classified as cytotoxic (all cytotoxics were non-personalized). More personalized drugs had the initiation of their clinical development (IND submission) during the contemporary period of time (2001–2010) compared to non-personalized drugs (75% of personalized drugs were developed between 2001 and 2010 while 42% of nonpersonalized drugs were developed in this time frame; *P* = .0186). All the remaining characteristics, including tumor type, year of approval, approved schedule (monotherapy vs. combination) and type of registration trial were not different between personalized and non-personalized drugs (Table 1).

**Table 1:** Comparison of personalized versus non-personalized drugs

Characteristic (%)	Personalized drugs (*N* = 28)	Non-personalized (*N* = 35)	*P*-Value
**Class of drug**			**< .0001**
**Cytotoxic**	0	14 (40)	
**Targeted**	28 (100)	21 (60)	
**Type of registration trial**			.618
**Randomized**	14 (50)	20 (57)	
**Non-randomized**	14 (50)	15 (43)	
**Treatment approved**			1.00
**Monotherapy**	24 (86)	30 (86)	
**Combination**	4 (14)	5 (14)	
**Type of tumor**			.321
**Solid**	14 (50)	22 (63)	
**Hematologic**	14 (50)	13 (37)	
Year of IND Submission*			**.0186**
**1981–2000**	7 (25)	19 (58)	
**2001–2010**	21 (75)	14 (42)	
**Year of approval**			.198
**1998–2006**	8 (29)	16 (46)	
**2007–2014**	20 (71)	19 (54)	
**Orphan drug program**			.107
**Yes**	22 (79)	20 (57)	
**No**	6 (21)	15 (43)	
**Priority review**			.772
**Yes**	22 (79)	26 (74)	
**No**	6 (21)	9 (26)	
**Fast track program**			.572
**Yes**	22 (79)	25 (71)	
**No**	6 (21)	10 (29)	
**Accelerated approval**			1.00
**Yes**	10 (36)	12 (34)	
**No**	18 (64)	23 (66)	

* two drugs were not included because IND submission was not available (decitabine and arsenic trioxide).

Abbreviations: IND, investigational new drug.

### Access to FDA special programs

In order to speed the development and availability of new drugs to treat serious conditions, the FDA has developed expedited programs, including the Priority Review, Fast Track Designation, Accelerated Approval and Breakthrough Therapy Designation. We evaluated the access of oncology drugs to at least one of these programs. Overall, of the 63 drugs included in our analysis only four drugs (axitinib, bosutinib, pazopanib and trametinib) had no access to at least one of these programs. Forty-eight drugs (76%) were granted Priority Review; 47(75%) received Fast Track designation; and 22(35%), Accelerated Approval. Breakthrough designation was created in July 2012, and hence only three approved drugs included in our database (ibrutinib, obinutuzumab, and ceritinib) were granted this designation. We compared the access to each of the FDA special programs between drugs developed under a personalized rationale versus non-personalized drugs (Table 1). No differences in access to these programs were detected between both groups.

The Orphan Drug Designation Program was created to offer special funding conditions for drugs intended to treat rare diseases, usually affecting less than 200,000 Americans, or when drugs were not expected to recover the costs of development. Overall 42 drugs (67%) received orphan drugs status. There was a trend for personalized drugs to received orphan drug designation more frequently (79% vs. 57% for non-personalized, *P* = .107).

### Personalized therapy was associated with faster clinical development

**Figure 2:**
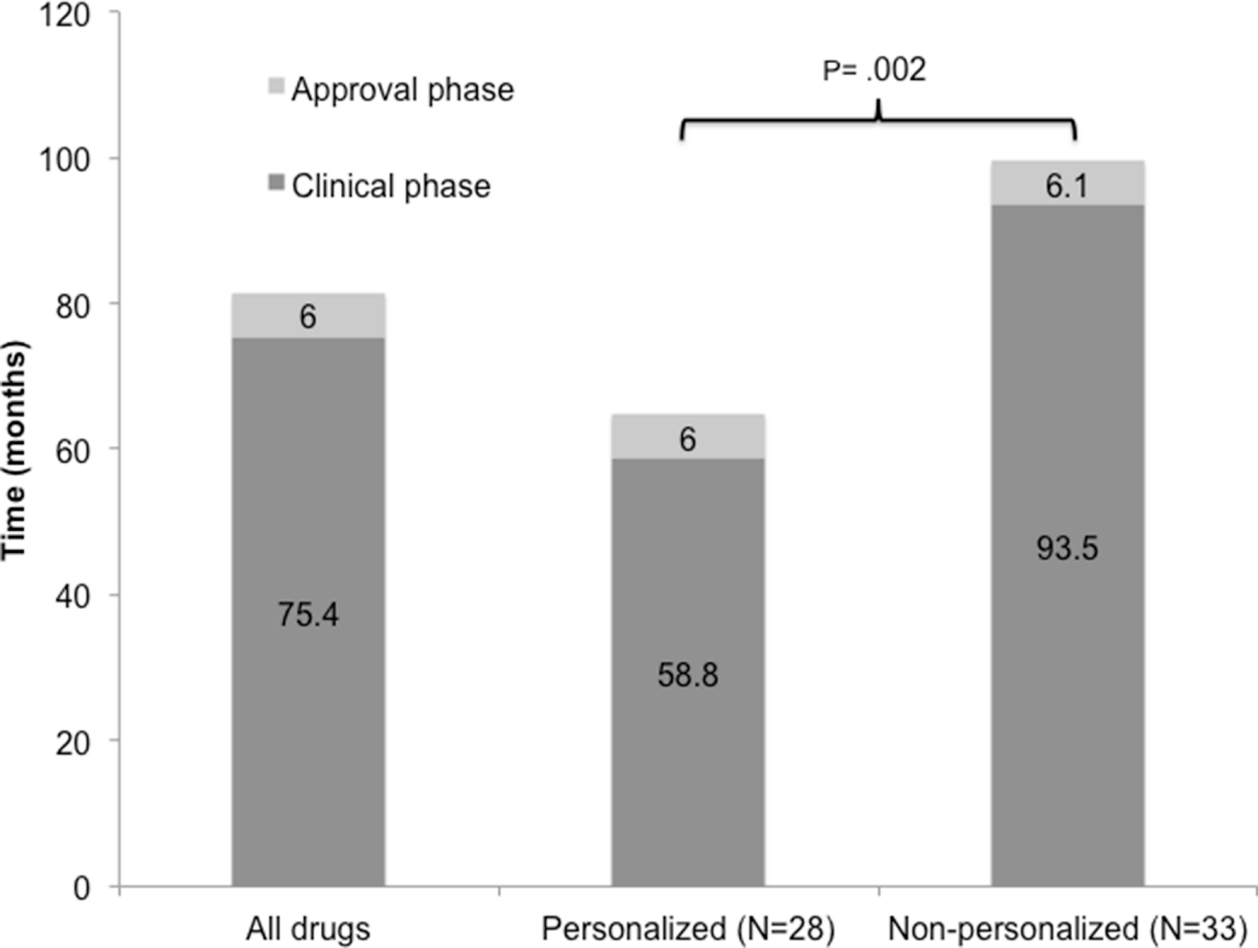
Development phases of drugs approved between 09/1998 and 07/2014 for the treatment of advanced cancers

Clinical and approval phase were calculated for included drugs (Supplementary Table S2). Arsenic trioxide and decitabine were included only on approval phase analysis due to absence of IND information. Median duration of clinical phase of development was 75.4 months (*N* = 61 drugs, 95% CI 67.2–94.8) and median approval time was 6.0 months (*N* = 63 drugs, 95% CI 6.0–7.8). Total time of clinical development (IND submission to NDA/BLA approval) took a median of 85.2 months (95% CI, 74.3–108.0). Drugs developed with a personalized strategy were associated with a faster clinical development phase (median =58.8 months [95% CI 53.9–81.8] vs. 93.5 months [95% CI 73.9–112.9] *P* = .001), but a similar duration of approval phase (median = 6.0 months [95% CI 5.5–8.4] vs. 6.1 months [95% CI 5.9–8.3], *P* = .756) when compared with non-personalized drugs, respectively (Figure 2). We also analyzed the influence of drug or registration trial characteristics upon approval times (Table 2). Although inclusion in an FDA special program (Fast Track or Accelerated Approval) was numerically associated with a shorter total time of development when compared with drugs not granted these conditions (decreases of 19.9 months and 6.4 months in median approval time, respectively) this was not statistically significant. Inclusion in a Priority review program was associated with a faster approval phase (6 months [95% CI 5.5–7.0] vs. 9.9 months [95% CI 9.7–11.5] for drugs with no priority, *P* = <.001); the total time of clinical development was shorter, albeit not statistically significant median = 77.5 versus 95 months; *P* = .41 (Table 2).

**Table 2:** Total time of drug development of FDA-approved anticancer agents stratified according to characteristics of interest

Parameters	N (drugs)	Total Clinical Development Time* Median (Months) (CI 95%)^a^	P (univariable)	P (multivariate)^b^
**Class of Drug**				
Targeted Biomarker-based selection (Personalized)	28	64.6 (59.5–86.7)	**.003**	**.038**
Targeted, No Biomarker-based selection (Non-personalized)	21	87.1 (74.3–116.2)		
Cytotoxic	12	112.7 (85.7–148.6)		
**Trial Design (registration trial)**				
Randomized	33	84.3 (75.2–108)	.919	—
Non-randomized	28	87.7 (64.3–112.1)		
**Treatment Approved**				
Monotherapy	52	86.0 (67.8–99.8)	.64	—
Combination	9	78.9 (68–127.4)		
**Type of Tumor**				
Solid	36	86.9 (75.3–111.8)	.34	—
ematologic	25	68.0 (60.8–111.4)		
**Year of IND Submission**				
1981–2000	26	109.2 (78.9–130.1)	**.006**	.07
2001–2010	35	67.8 (62.0–87.8)		
**Year of Approval**				
1998–2006	22	78.2 (67.8–110.4)	.499	—
2007–2014	39	88.9 (69.2–115.6)		
**Orphan Drug Program**				
Yes	40	80.3 (62.2–112.4)	.316	—
No	21	86.7 (77.5–111.8)		
**Fast Track Program**				
Yes	45	79.8 (67.7–99.1)	.294	—
No	16	99.7 (75.3–124)		
**Accelerated Approval**				
Yes	22	80.3 (62.2–112.3)	.764	—
No	39	86.7 (75.8–99.8)		
**Priority Review**				
Yes	47	77.5 (67.5–99.8)	.41	—
No	14	95.0 (79.8–122.6)		

* : total clinical development time was defined as the sum of clinical (IND to NDA submission) and approval time (NDA submission to approval).

a : two non-personalized drugs were excluded from Total Approval Time analysis because IND data was not available (arsenic trioxide and decitabine).

b : only significant factors on univariate analysis were included in the multivariate model.

In the multivariate analysis, the class of drug (targeted vs. cytotoxic) stratified by the development of a drug under a personalized therapy strategy remained as the only independent factor associated with a faster drug development timeline (median = 64.6 (personalized targeted) vs 87.1 (targeted non-personalized) vs. 112.7 months (cytotoxic) (*P* = .038) (Table 2).

### Response rates (RR) in early trials and drug approval time

**Figure 3:**
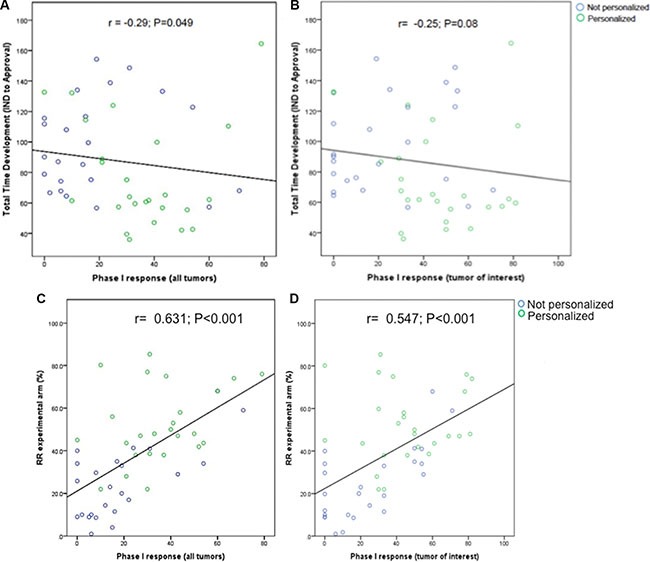
Reported response rates in phase I trials in all tumors (A) and tumors in which the drug was eventually approved (B) and total time of drug development (IND submission to NDA/BLA approval) in months Total time of development was inversely associated with response rate. Reported response rates in phase I trials in all tumors (**C**) and tumors in which the drugs were eventually approved (**D**) and response rates in registration trials of the same drug. Phase I trials were matched to registration trials as described in methods section and previously published. [31].

We investigated whether the RR in phase I trials of FDA-approved agents was correlated with total time of clinical development. RR in all tumors included in a phase I was inversely correlated with total time of drug development (*r* = −0.29, *P* = .049, Figure 3A). A similar correlation was detected when analyzing only the RR in the tumor of interest (which subsequently received FDA approval for the drug), although not statistically significant (*r* = −0.25, *P* = .08, Figure 3B). We further investigated the correlation between RR in early trials and RR in the registration trial for each drug. Both RR in all tumors (*r* = 0.631, *P* < .001, Figure 3C) and RR in the tumor of interest (*r* = 0.547, *P* < .001, Figure 3D) were found to positively correlate with RR in the respective registration trial of the drug.

## DISCUSSION

One of the greatest challenges in oncology is to deliver more efficient and safer drugs in a shorter time frame to patients with cancer. Our analysis of novel anticancer agents approved by the FDA between 09/1998 and 07/2014 showed that drugs developed under a biomarker-based (also known as personalized or precision oncology) strategy were associated with a significantly shorter clinical phase of development (median = 58.8 months vs. 93.5 months for non-personalized drugs, *P* = .001). Although FDA special programs hold the promise to speed the development program, the strategy for the development of a cancer drug was still the most important factor to influence this process. Our multivariate analysis revealed that targeted agents were developed significantly faster when there was a biomarker-based selection approach than when targeted agents were given to unselected patients or as compared to cytotoxic agents (*P* = .038).

A previous study by DiMasi and Grabowski [13] reported that oncology drugs have more access to FDA special programs than non-oncology drugs. Consequently, median approval time (NDA/BLA submission to final approval) was shorter for oncology drugs (1.0 vs. 1.3 years). However, the authors described a longer clinical phase of development for these drugs, compared to non-oncology agents (7.8 vs. 6.3 years). The current oncology drug development model is characterized by high attrition rates and a substantial lag time between different steps [8, 13] due to multiple factors, including regulatory burden and difficulty with designing definitive trials [14]. In addition, drugs have been traditionally developed for cancer populations selected solely by histology. In order to obtain regulatory approval, large trials were designed to show a small, but statistical significant clinical benefit in large groups [15]. As a consequence, longer clinical development, higher costs, and a high failure rate of oncology drugs have led to doubts regarding the sustainability of this approach for drug development [8, 9].

Recently, efforts were made to improve the drug development model and to reduce the failure rate of oncology drugs, as well as to shorten the time for drug development [5]. The recognition of molecular abnormalities that characterize certain tumor types led to the development of more specific targeted agents, aiming to be more efficient in a biomarker-positive population [16]. Consequently, incorporation of a biomarker-driven strategy has been encouraged by some regulatory agencies [1, 5]. Not surprisingly, our results demonstrated a reasonable number of personalized drugs (44%) among anticancer agents approved by the FDA since 1998. In addition, over the more recent period of approvals (from 2007 to 2014), drugs developed with a biomarker-based rationale outnumbered those without such a rationale (20 vs. 19 drugs).

It is well recognized that biomarker development is also challenging. Inter and intratumoral heterogeneity can compromise the widespread use of genetic and protein biomarkers; technical challenges include the need for analytical validation of tests and also finding samples that are more feasible to be used; and clinical validation of a predictive test often needs prospective studies [17]. Several practical strategies to facilitate biomarker development have been proposed, including optimization of pre-clinical models, early standardization of tests and close mirroring of clinical drug development with biomarker development [18]. This early introduction of biomarker development during a drug development timeline can improve the success, including a faster and more efficient model.

The FDA has also expanded its expedited programs to facilitate drug development. In agreement with previous data [13], we report a high rate of access to Priority Review (76%), Fast Track Designation (75%) and Accelerated Approval (35%) for oncology drugs included in our database. Although we could not detect differences in access to these programs between personalized and non-personalized drugs, a trend for receiving more orphan drug status (79% vs. 57%, *P* = .107) was characteristic of personalized drugs. We could not discern statistically significant differences in time to approval for drugs that had access to any one of the special FDA programs, though each had a numerically shorter time to approval (Table 2). Our finding that even accelerated approval was not associated with a shorter development time mirrors the findings from a prior study [12]. It is possible that drugs chosen for accelerated approval pathway target a more restricted cancer population. Thus, slow trial accrual may counterbalance the benefit of approval based on surrogate endpoints upon development timeline. Further, only four drugs had no access to any special program; therefore determining the effect of lack of access to special programs was not possible. Of note, Priority Review Status did shorten the time from NDA/BLA submission to final approval (median = 9.9 to 6.0 months; *P* < .001); overall median time from IND submission to approval was 77.5 versus 95 months; *P* = .27. It is important to note that only three drugs (ibrutinib, obinutuzumab and ceritinib –all personalized) were approved under the newly minted Breakthrough Designation, and, therefore, more experience with this mechanism is needed to determine its impact.

Targeted drugs that were developed for a molecularly selected cancer population had some of the shortest clinical development timelines. Examples are imatinib for chronic myelogenous leukemia (CML; Bcr/Abl translocation) (total clinical phase time = 36 months), ceritinib for ALK-positive non-small cell lung cancer (total clinical phase time = 42.7 months; approved after Phase I) and dabrafenib for BRAF-positive melanoma (total clinical development time = 47.1 months). All these drugs had access to at least one FDA special program. Aiming to obtain a large difference in a small patient group was an important driver of their success. It is possible that a higher clinical activity of biomarker driven drugs, as previously demonstrated [7, 19, 20], could also explain their faster clinical development. It is also interesting to note that we could not detect differences in total approval time if the drug approval was based on a randomized or non-randomized trial (Table 2). Approving drugs based on non-randomized trials was previously also demonstrated to be safe [21].

Our analysis of drug development timelines was restricted to the FDA database. It would be necessary to determine if these results could be extrapolated to other regulatory agencies (such as European Medicines Agency and Health Canada), which could face similar regulatory timelines [22]. The requirements for the development process of molecularly-oriented treatments are not well established for many agencies, and the co-development of companion diagnostic tests and access to expedited programs is a paradigm that is still evolving [23, 24]. A global effort to harmonize the guidelines for the development of personalized therapies may assist international trials that could be needed for ultra-rare populations.

Not surprisingly, we also showed that a higher response rate in early trials correlated with shorter drug development times. Moreover, responses in early phase trials were positively correlated to responses in registration trials. Since we did not include agents that failed to obtain FDA approval, we cannot establish whether or not early responses are a surrogate of drug success. Even so, activity appears to be an important endpoint for phase I trials. Indeed, high activity in dose expansions of phase I trials led to easier access to FDA expedited programs and notably shorter approval times for crizotinib and ceritinib [11, 25]. Both ceritinib (ALK inhibitor) and more recently pembrolizumab (anti-PD-1) have been approved by the FDA after Phase I testing [11, 26].

The approval of trastuzumab for Her-2 positive, advanced breast cancer was an important factor in triggering an era of biomarker-guided drug development. Advances in molecular diagnostic and research techniques are leading to more widespread acceptance of the concept of precision oncology. Herein, we demonstrate that the strategies that underlie precision oncology can also hasten the clinical phase of drug development and time to approval of new agents.

## MATERIALS AND METHODS

### Search strategy

To analyze various aspects of drug development in oncology after the introduction of biomarker-based therapies, we reviewed agents newly approved for the treatment of advanced cancer by the FDA between September 25^th^ 1998 (FDA approval of trastuzumab for metastatic Her2 positive breast cancer) and July 3rd, 2014. New molecular entities' (NMEs) approvals, package inserts and review documents were obtained for analysis through the FDA website [27]. When first investigational new drug (IND) application information was not fully available in the FDA documents, we also consulted the Federal Register website for regulatory review process documents related to patent restoration [28]. Information about drug approvals and access to FDA expedited programs were also compared with contemporary literature [29]. Information about registration trials was obtained from the FDA original package inserts and original publications were obtained through MEDLINE or ASCO meetings' website [30] (if the clinical trial was not yet published). We concomitantly searched MEDLINE for phase I trials for each of the agents selected from the FDA database analysis. These trials were matched with registration trials of the respective agent if they evaluated a non-pediatric cancer patients and explored either monotherapy (as FDA-approved) or the same combination and schedule as described in the FDA package insert; in addition, the phase I trial should have started before the later clinical trial and included similar patient populations of solid versus hematologic malignancies [31]. We excluded agents approved for pediatric cancer, supportive care, loco-regional treatment, hormonal therapies, vaccines and agents that already had been approved before search period or discontinued in the US market.

### Data extraction

Data extraction was conducted independently by two investigators (DFJ and MS) and any discrepancies were resolved by consensus in frequent meetings in the presence of the principal investigator (RK). As for drug development information, we obtained data of IND submission, first new drug application (NDA) or biologic license application (BLA) submission and approval, and access to FDA expedited programs (Supplementary Table S2). We considered US clinical phase as the time between first IND submission and NDA/BLA submission, and approval phase as the time of first NDA/BLA submission to approval [13]. Total clinical development time was considered as the sum of clinical and approval phases.

From the clinical trials, we obtained information about trial characteristics and efficacy, described as response rates (RRs). Although responses are not the main outcome considered by the FDA for drug approval, they are available from phase I trials and could indicate early activity [32]. Responses were recorded according to the response criteria adopted in the trial: for solid tumors, partial and complete responses as per Response Evaluation in Solid Tumors Criteria (RECIST) [33] or World Health Organization Criteria (WHO) [34]; for chronic myelogenous leukemia (CML), major cytogenetic responses; for multiple myeloma (MM), partial and complete responses; for acute myelogenous leukemia (AML), complete responses; and for lymphomas, partial and complete responses by WHO criteria. We captured responses in phase I trials for all cancer patients and for patients with the cancer type that subsequently led to FDA approval (tumor of interest). Lack of standardization for reporting RR in phase I trials precluded the analysis of dose levels upon responses.

For the purpose of our analysis, personalized therapy, biomarker-based strategy, and precision oncology were used interchangeably. We considered a therapy as “personalized” when it met one of the following criteria:

- Cognate biomarker used to select patients for treatment OR
- No cognate biomarker used, but at least 50% of patients are known to harbor a cognate biomarker.

We classified as targeted, agents designed to specifically impact signals in the cancer cell that are abnormal or are differentially expressed compared to those in normal elements.

### Statistical analysis

Assessment of independent samples was done using a Wilcoxon rank-sum test. The multiple linear regression model for approval time contained only the independent variables that were significant in the univariate analysis. Categorical data were compared using Fisher's exact test. The correlations between response rate and approval time were estimated using Spearman rank correlation coefficient. A *P* value of .05 or less was considered statistically significant. Statistical analyses were done by MS using SPSS version 22 (SPSS) software.

## SUPPLEMENTARY MATERIAL TABLES





## References

[1] Cancer Genome Atlas Research N (2012). Comprehensive genomic characterization of squamous cell lung cancers. Nature.

[2] Cancer Genome Atlas Research N (2014). Comprehensive molecular profiling of lung adenocarcinoma. Nature.

[3] Hutchinson KE, Lipson D, Stephens PJ, Otto G, Lehmann BD, Lyle PL, Vnencak-Jones CL, Ross JS, Pietenpol JA, Sosman JA, Puzanov I, Miller VA, Pao W (2013). BRAF fusions define a distinct molecular subset of melanomas with potential sensitivity to MEK inhibition. Clin Cancer Res.

[4] Krampitz GW, Norton JA (2014). RET gene mutations (genotype and phenotype) of multiple endocrine neoplasia type 2 and familial medullary thyroid carcinoma. Cancer.

[5] Food and Drug Administration and US Department of Health and Human Services (2006). Critical Path Report.

[6] Sharma MR, Schilsky RL (2012). Role of randomized phase III trials in an era of effective targeted therapies. Nat Rev Clin Oncol.

[7] Fontes Jardim DL, Schwaederle M, Wei C, Lee JJ, Hong DS, Eggermont AM, Schilsky RL, Mendelsohn J, Lazar V, Kurzrock R (2015). Impact of a Biomarker-Based Strategy on Oncology Drug Development: A Meta-analysis of Clinical Trials Leading to FDA Approval. J Natl Cancer Inst.

[8] Rubin EH, Gilliland DG (2012). Drug development and clinical trials—the path to an approved cancer drug. Nat Rev Clin Oncol.

[9] Jonsson B, Bergh J (2012). Hurdles in anticancer drug development from a regulatory perspective. Nat Rev Clin Oncol.

[10] Gerber DE, Minna JD (2010). ALK inhibition for non-small cell lung cancer: from discovery to therapy in record time. Cancer Cell.

[11] Dhillon S, Clark M (2014). Ceritinib: first global approval. Drugs.

[12] Richey EA, Lyons EA, Nebeker JR, Shankaran V, McKoy JM, Luu TH, Nonzee N, Trifilio S, Sartor O, Benson AB, Carson KR, Edwards BJ (2009). Accelerated approval of cancer drugs: improved access to therapeutic breakthroughs or early release of unsafe and ineffective drugs?. J Clin Oncol.

[13] DiMasi JA, Grabowski HG (2007). Economics of new oncology drug development. J Clin Oncol.

[14] Stewart DJ, Whitney SN, Kurzrock R (2010). Equipoise lost: ethics, costs, and the regulation of cancer clinical research. J Clin Oncol.

[15] Stewart DJ, Kurzrock R (2009). Cancer: the road to Amiens. J Clin Oncol.

[16] Braiteh F, Kurzrock R (2007). Uncommon tumors and exceptional therapies: paradox or paradigm?. Mol Cancer Ther.

[17] Meric-Bernstam F, Mills GB (2012). Overcoming implementation challenges of personalized cancer therapy. Nat Rev Clin Oncol.

[18] de Gramont A, Watson S, Ellis LM, Rodon J, Tabernero J, de Gramont A, Hamilton SR (2015). Pragmatic issues in biomarker evaluation for targeted therapies in cancer. Nat Rev Clin Oncol.

[19] Fontes Jardim DL, Schwaederle MC, Lee JJ, Hong DS, Eggermont AM, Schilsky RL, Mendelsohn J, Lazar V, Kurzrock R (2014). Systematic review of a personalized strategy in cancer clinical trials leading to FDA approval. ASCO Meeting Abstracts.

[20] Janku F, Berry DA, Gong J, Parsons HA, Stewart DJ, Kurzrock R (2012). Outcomes of phase II clinical trials with single-agent therapies in advanced/metastatic non-small cell lung cancer published between 2000 and 2009. Clin Cancer Res.

[21] Tsimberidou AM, Braiteh F, Stewart DJ, Kurzrock R (2009). Ultimate fate of oncology drugs approved by the us food and drug administration without a randomized Trial. J Clin Oncol.

[22] Downing NS, Aminawung JA, Shah ND, Braunstein JB, Krumholz HM, Ross JS (2012). Regulatory review of novel therapeutics–comparison of three regulatory agencies. N Engl J Med.

[23] Senderowicz AM, Pfaff O (2014). Similarities and differences in the oncology drug approval process between FDA and European Union with emphasis on *in vitro* companion diagnostics. Clin Cancer Res.

[24] Hirsch FR, Bunn PA (2014). and Herbst RS. “Companion diagnostics”: has their time come and gone? Clin Cancer Res.

[25] Gandhi L, Janne PA (2012). Crizotinib for ALK-rearranged non-small cell lung cancer: a new targeted therapy for a new target. Clin Cancer Res.

[26] Poole RM (2014). Pembrolizumab: first global approval. Drugs.

[27] Food and Drug Administration (FDA) Drugs @FDA.

[28] Federal Register https://www.federalregister.gov/articles/search.

[29] Kesselheim AS, Darrow JJ (2014). Drug development and FDA approval, 1938–2013. N Engl J Med.

[30] American Society of Clinical Oncology meeting website http://meetinglibrary.asco.org/abstracts.

[31] Jardim DL, Hess KR, Lorusso P, Kurzrock R, Hong DS (2014). Predictive value of phase I trials for safety in later trials and final approved dose: analysis of 61 approved cancer drugs. Clin Cancer Res.

[32] Sekine I, Yamamoto N, Kunitoh H, Ohe Y, Tamura T, Kodama T, Saijo N (2002). Relationship between objective responses in phase I trials and potential efficacy of non-specific cytotoxic investigational new drugs. Ann Oncol.

[33] Therasse P, Arbuck SG, Eisenhauer EA, Wanders J, Kaplan RS, Rubinstein L, Verweij J, Van Glabbeke M, van Oosterom AT, Christian MC, Gwyther SG (2000). New guidelines to evaluate the response to treatment in solid tumors. European Organization for Research and Treatment of Cancer, National Cancer Institute of the United States, National Cancer Institute of Canada. J Natl Cancer Inst.

[34] Miller AB, Hoogstraten B, Staquet M, Winkler A (1981). Reporting results of cancer treatment. Cancer.

