# Supplementary material for: An appraisal of drug development timelines in the Era of precision oncology
Source: Oncotarget. 2016 Jul 13;7(33):53037–46. doi: 10.18632/oncotarget.10588 (PMC5288167; doi:10.18632/oncotarget.10588)
Supplement: Supplementary file 1 [file oncotarget-07-53037-s001.pdf]

An appraisal of drug development timelines in the Era of precision oncology

Supplementary Materials

Supplementary Table S1: Description of FDA special access programs for drug development

Program	Description	Start
**Orphan Drug Designation**	Provides a special status for a drug or biological intended to treat a rare disease. Qualifies the sponsor for development incentives, including tax credits.	Jan/1983
**Fast Track**	Designed to facilitate the development and review of drugs to treat serious and unmet needs. FDA offers more frequent meetings and written communication for Fast Track drugs and a rolling review for NDA/BLA application.	Nov/1997
**Accelerated approval**	Allows FDA to approve promising therapies based on a positive effect upon "surrogate endpoints" that are likely to predict clinical benefit. After approval, the sponsor may be required to conduct post-marketing trials to confirm benefits.	Oct/1992
**Priority Review**	Designed to expedite the approval phase of new molecular entities from the standard 10 months to less than 6 months. The designation is determined at the time of BLA or NDA application.	Oct/1992
**Breakthrough Designation**	Intended to expedite the development and review of drugs for serious or life-threatening conditions. Includes all fast track program features plus more intensive FDA guidance. Requires preliminary clinical evidence demonstrating substantial improvement over existing therapies.	Jul/2012

BLA: biologic license application; NDA: new drug application.

Supplementary Table S2: List of included drugs and their respective timeline information, including access to FDA special programs. See Supplementary_Table_S2

Supplementary Table S3: Classification of drugs according to the criteria used to define a biomarker-driven treatment. See Supplementary_Table_S3
