# Supplementary material for: An appraisal of drug development timelines in the Era of precision oncology
Source: Oncotarget. 2016 Jul 13;7(33):53037–46. doi: 10.18632/oncotarget.10588 (PMC5288167; doi:10.18632/oncotarget.10588)
Supplement: Supplementary file 2 [file oncotarget-07-53037-s002.docx]

**Supplemental Table 2 – List of included drugs and their respective timeline information, including access to FDA special programs**

| Drug | | IND | NDA/BLA submission | | FDA approval | Clinical Phase (months) | Approval Phase (months) | | FDA Program |
| --- | --- | --- | --- | --- | --- | --- | --- | --- | --- |
| Personalized therapies (N=28)* | | | | | | | | |  |
| Afatinib | | 12/31/03 | | 11/14/12 | 7/12/13 | 106.47 | | 7.93 | F,O,P |
| Alemtuzumab | | 12/25/91 | | 12/23/99 | 5/7/01 | 95.93 | | 16.47 | A,F,O |
| Bosutinib | | 5/9/04 | | 11/17/11 | 9/4/12 | 90.27 | | 9.57 | O |
| Brentuximab vedotin | | 6/27/06 | | 2/25/11 | 8/19/11 | 55.93 | | 5.80 | A,F,O,P |
| Cabozantinib | | 7/1/05 | | 5/21/12 | 11/29/12 | 82.67 | | 6.27 | F,O,P |
| Ceritinib | | 10/08/10 | | 12/24/13 | 4/29/14 | 38.53 | | 4.17 | A,B,F,O,P |
| Crizotinib | | 1/12/06 | | 3/30/11 | 8/26/11 | 62.60 | | 4.87 | A,F,O,P |
| Dabrafenib | | 6/26/09 | | 7/29/12 | 5/29/13 | 37.10 | | 10.00 | F,O |
| Dasatinib | | 3/11/03 | | 12/28/05 | 6/28/06 | 33.57 | | 6.00 | F,O,P |
| Ibrutinib | | 9/08/08 | | 6/28/13 | 11/13/13 | 57.67 | | 4.5 | A,B,F,O,P |
| Ibritumomab | | 12/7/92 | | 11/1/00 | 2/19/02 | 94.80 | | 15.60 | A,F,O |
| Imatinib | | 5/10/98 | | 2/27/01 | 5/10/01 | 33.57 | | 2.43 | A,O,P |
| Lapatinib | | 12/6/00 | | 9/13/06 | 3/13/07 | 69.23 | | 6.00 | F,P |
| Nilotinib | | 4/24/04 | | 9/29/06 | 10/29/07 | 29.17 | | 13.00 | A,F,O |
| Obinutuzumab | | 2/06/09 | | 4/22/13 | 11/01/13 | 50.53 | | 6.30 | B,F,O,P |
| Ofatumumab | | 5/20/04 | | 1/30/09 | 10/26/09 | 56.33 | | 8.87 | A,F,O,P |
| Pertuzumab | | 6/1/01 | | 12/8/11 | 6/8/12 | 126.23 | | 6.00 | P |
| Ponatinib | | 11/21/07 | | 9/27/12 | 12/14/12 | 58.20 | | 2.57 | A,F,O,P |
| Ruxolitinib | | 3/30/07 | | 6/3/11 | 11/16/11 | 50.10 | | 5.43 | F,O,P |
| Siltuximab | | 12/22/03 | | 08/30/13 | 4/22/14 | 116.27 | | 7.73 | P,O |
| Sunitinib | | 4/16/01 | | 8/11/05 | 1/26/06 | 51.83 | | 5.50 | F,P |
| Tositumomab | | 10/13/89 | | 9/14/00 | 6/27/03 | 131.03 | | 33.43 | F,O,P |
| Trametinib | | 4/14/08 | | 8/3/12 | 5/29/13 | 51.63 | | 9.87 | O |
| Trastuzumab | | 4/11/92 | | 5/4/98 | 9/25/98 | 72.77 | | 4.70 | F,P |
| Trastuzumab emtansine | | 12/1/05 | | 8/27/12 | 2/22/13 | 80.87 | | 5.83 | F,P |
| Vandetanib | | 3/16/00 | | 7/7/10 | 4/6/11 | 123.70 | | 8.97 | F,O,P |
| Vemurafenib | | 9/1/06 | | 4/28/11 | 8/17/11 | 55.90 | | 3.63 | F,O,P |
| Vismodegib | | 9/29/06 | | 9/8/11 | 1/30/12 | 59.30 | | 4.73 | P |
| Non-personalized drugs (N=35) | | | | | | | | |  |
| Arsenic trioxide | N/A | | 3/27/00 | | 9/25/00 | N/A | 5.93 | | F,O,P |
| Axitinib | 11/9/01 | | 4/14/11 | | 1/27/12 | 113.17 | 9.43 | |  |
| Belinostat | 11/15/04 | | 12/08/13 | | 07/03/14 | 108.77 | 6.83 | | A,F,O,P |
| Bendamustine | 6/11/03 | | 9/19/07 | | 3/20/08 | 51.27 | 6.03 | | O,P |
| Bevacizumab | 7/31/97 | | 9/26/03 | | 2/26/04 | 73.87 | 5.00 | | F,P |
| Bortezomib | 8/22/98 | | 1/21/03 | | 5/13/03 | 52.97 | 3.73 | | A,F,O,P |
| Cabazitaxel | 4/14/99 | | 3/31/10 | | 6/17/10 | 131.57 | 2.57 | | F,P |
| Carfilzomib | 6/13/05 | | 9/26/11 | | 7/20/12 | 75.43 | 9.80 | | A,F,O |
| Cetuximab | 10/18/94 | | 8/14/03 | | 2/12/04 | 105.87 | 5.93 | | A,F,P |
| Decitabine | N/A | | 11/14/05 | | 5/2/06 | N/A | 5.60 | | F,O |
| Denosumab | 5/21/01 | | 12/11/12 | | 6/13/13 | 138.67 | 6.07 | | P |
| Eribulin | 3/31/03 | | 3/30/10 | | 11/15/10 | 84.00 | 7.50 | | F,P |
| Erlotinib | 8/16/97 | | 7/30/04 | | 11/18/04 | 83.47 | 3.60 | | F,P |
| Everolimus | 11/22/02 | | 6/27/08 | | 3/30/09 | 67.17 | 9.10 | | P |
| Ipilimumab | 8/12/00 | | 6/25/10 | | 3/25/11 | 118.43 | 9.00 | | F,P,O |
| Ixabepilone | 6/30/99 | | 4/16/07 | | 10/16/07 | 93.53 | 6.00 | | P |
| Lenalidomide | 4/27/00 | | 4/7/05 | | 12/27/05 | 59.33 | 8.67 | | F,P,O |
| Nelarabine | 6/9/93 | | 4/29/05 | | 10/28/05 | 142.67 | 5.97 | | A,F,P,O |
| Omacetaxine | 7/1/81 | | 3/30/12 | | 10/26/12 | 368.97 | 6.87 | | A,O |
| Oxaliplatin | 4/3/93 | | 6/24/02 | | 8/9/02 | 110.70 | 1.50 | | A,F,P |
| Paclitaxel protein-bound | 5/12/98 | | 3/8/04 | | 1/7/05 | 69.87 | 9.97 | | F |
| Panitumumab | 2/2/01 | | 3/29/06 | | 9/27/06 | 61.90 | 5.93 | | A,F,P |
| Pazopanib | 10/10/02 | | 12/19/08 | | 10/19/09 | 74.30 | 10.00 | |  |
| Pemetrexed | 7/8/92 | | 9/30/03 | | 2/4/04 | 134.73 | 4.13 | | F,O,P |
| Pomalidomide | 11/13/02 | | 4/10/12 | | 2/8/13 | 112.90 | 9.93 | | A,O |
| Pralatrexate | 8/17/98 | | 3/24/09 | | 9/24/09 | 127.23 | 6.00 | | A,F,O,P |
| Ramucirumab | 07/29/04 | | 8/23/13 | | 4/21/14 | 108.8 | 7.93 | | F,O,P |
| Regorafenib | 7/19/06 | | 4/27/12 | | 9/27/12 | 69.27 | 5.00 | | F,P |
| Romidepsin | 4/30/02 | | 1/12/09 | | 11/5/09 | 80.40 | 9.77 | | F,O |
| Sorafenib | 5/30/00 | | 7/8/05 | | 12/20/05 | 61.27 | 5.40 | | F,O,P |
| Temozolomide | 5/3/93 | | 8/13/98 | | 8/11/99 | 63.33 | 11.93 | | A,O,P |
| Temsirolimus | 5/29/98 | | 10/5/06 | | 5/30/07 | 100.20 | 7.83 | | F,O,P |
| Vincristine liposomal | 9/30/99 | | 7/12/11 | | 8/9/12 | 141.40 | 12.90 | | A,F,O |
| Vorinostat | 5/21/01 | | 4/7/06 | | 10/6/06 | 58.53 | 5.97 | | F,O,P |
| Ziv-aflibercept | 8/2/01 | | 10/28/11 | | 8/3/12 | 122.87 | 9.17 | | P |

Abbreviations: A, accelerated approval; B, breakthrough therapy designation; BLA, biologic license application; IND, investigational new drug; F, fast track program; NDA, new drug application; O, orphan status designation; P, priority review. *Drugs were considered personalized when they met one of the following criteria: a. Cognate biomarker used to select patients for treatment OR

b. No cognate biomarker used, but at least 50% of patients are known to

harbor a cognate biomarker.

**Supplemental Table 3 – Classification of drugs according to the criteria used to define a biomarker-driven treatment**

| Biomarker tested  *N*=17  (*Target*) | Rationale |
| --- | --- |
| Afatinib  (*EGFR*) | EGFR mutation testing required |
| Bosutinib  (*Bcr-Abl tyrosine kinase*) | Philadelphia chromosome testing required |
| Ceritinib  (*ALK*) | ALK rearrangement testing required |
| Crizotinib  (*ALK*) | ALK rearrangement testing required |
| Dabrafenib  (*BRAF*) | BRAF mutation testing required |
| Dasatinib  (*Bcr-Abl tyrosine kinase*) | Philadelphia chromosome testing required |
| Imatinib  (*Bcr-Abl tyrosine kinase*) | Philadelphia chromosome testing required |
| Lapatinib  (*Her-2*) | Her-2 expression testing required |
| Nilotinib  (*Bcr-Abl tyrosine kinase*) | Philadelphia chromosome testing required |
| Pertuzumab  (*Her-2*) | Her-2 expression testing required |
| Ponatinib  (*Bcr-Abl tyrosine kinase*) | Philadelphia chromosome testing required |
| Sunitinib  (*c-Kit*) | c-Kit expression testing required |
| Tositumumab  (*CD20*) | CD20 expression testing required |
| Trametinib  (*MEK*) | BRAF mutation testing required. BRAF mutation leads to MEK activation |
| Trastuzumab  (*Her-2*) | Her-2 expression testing required |
| Trastuzumab Entansine  (*Her-2*) | Her-2 expression testing required |
| Vemurafenib  (*BRAF*) | BRAF mutation testing required |
| Putative Presence of Cognate Biomarker  *N*=11  *(Target)* | **Rationale** |
| Alemtuzumab  (*CD52*) | CD52 is a characteristic of B-cell leukemic cells and is targeted by the drug |
| Brentuximab  (*CD30*) | CD30 expression is present in Hodgkin`s Lymphoma cells |
| Cabozantinib  (*RET*) | More than 50% of patients with medullary thyroid cancer are known to harbor RET mutations |
| Ibrutinib  (*Burton`s tyrosine kinase*) | Bruton`s tyrosine kinase is an essential component of the B-cell receptor signaling pathway |
| Ibritumomab  (*CD20*) | The CD20 antigen is expressed on pre-B and mature B lymphocytes and on > 90% of B-cell non-Hodgkin’s lymphomas |
| Obinotuzumab  (*CD20*) | CD20 is constitutively expressed on the surface of CLL cells |
| Ofatumumab  (*CD20*) | CD20 is constitutively expressed on the surface of CLL cells |
| Ruxolitinib  (*JAK1/JAK2*) | Myelofibrosis is a myeloproliferative neoplasm known to be associated with deregulated JAK1 and JAK2 signaling. Cosmic database describes 57% of patients as having JAK2 mutations. |
| Siltuximab  (*IL-6*) | Up regulation of IL-6 production is a hallmark of Castleman`s disease development |
| Vandetanib  (*RET*) | More than 50% of medullary thyroid cancer patients harbor RET mutations |
| Vismodegib  (*Hedgehog pathway*) | Loss of function of patched homologue 1 (PTCH1), which normally acts to inhibit the signaling activity of smoothened homologue (SMO). Reported rates: 12% mutation SMO and 39% PTCH1 = 51 % hedgehog pathway mutations in basal cell carcinoma |
